# Granulocyte Colony-Stimulating Factor Treatment Before Radiotherapy Protects Against Radiation-Induced Liver Disease in Mice

**DOI:** 10.3389/fphar.2021.725084

**Published:** 2021-11-15

**Authors:** Isalira Peroba Rezende Ramos, Marlon Lemos Dias, Alan Cesar Nunes De Moraes, Fernanda Guimarães Meireles Ferreira, Sergio Augusto Lopes Souza, Bianca Gutfilen, Thiago Barboza, Cibele Ferreira Pimentel, Cintia Marina Paz Batista, Tais Hanae Kasai-Brunswick, Fabio Da Silva De Azevedo Fortes, Cherley Borba Vieira De Andrade, Regina Coeli dos Santos Goldenberg

**Affiliations:** ^1^ Centro Nacional de Biologia Estrutural e Bioimagem-CENABIO, Universidade Federal do Rio de Janeiro, UFRJ, Rio de Janeiro, Brazil; ^2^ Instituto de Biofísica Carlos Chagas Filho, Universidade Federal do Rio de Janeiro, UFRJ, Rio de Janeiro, Brazil; ^3^ Instituto Nacional de Ciência e Tecnologia em Medicina Regenerativa, INCT-REGENERA, Universidade Federal do Rio de Janeiro, UFRJ, Rio de Janeiro, Brazil; ^4^ Departamento de Biologia, Universidade Federal Fluminense, UFF, Niterói, Brazil; ^5^ Instituto D’Or de Pesquisa e Educação, Rio de Janeiro, Brazil; ^6^ Departamento de Radiologia, Hospital Universitário Clementino Fraga Filho, Universidade Federal do Rio de Janeiro, Rio de Janeiro, Brazil; ^7^ Laboratório de Terapia e Fisiologia Celular e Molecular-LTFCM, Centro Universitário Estadual da Zona Oeste-UEZO, Rio de Janeiro, Brazil; ^8^ Programa de Pós-Graduação em Biomedicina Translacional-BIOTRANS (UEZO-UNIGRANRIO-InMETRO), Duque de Caxias, Brazil; ^9^ Departmento de Histologia e Embriologia, Universidade do Estado do Rio de Janeiro, UERJ, Rio de Janeiro, Brazil

**Keywords:** liver, irradiation, immunotherapy, G-CSF, alcohol, ultrasonography, magnetic resonance imaging, computed tomography

## Abstract

Radiation-induced liver disease (RILD) remains a major problem resulting from radiotherapy. In this scenario, immunotherapy with granulocyte colony-stimulating factor (G-CSF) arises as an attractive approach that might improve the injured liver. Here, we investigated G-CSF administration’s impact before and after liver irradiation exposure using an association of alcohol consumption and local irradiation to induce liver disease model in C57BL/6 mice. Male and female mice were submitted to a previous alcohol-induced liver injury protocol with water containing 5% alcohol for 90 days. Then, the animals were treated with G-CSF (100 μg/kg/d) for 3 days before or after liver irradiation (18 Gy). At days 7, 30, and 60 post-radiation, non-invasive liver images were acquired by ultrasonography, magnetic resonance, and computed tomography. Biochemical and histological evaluations were performed to verify whether G-CSF could prevent liver tissue damage or reverse the acute liver injury. Our data showed that the treatment with G-CSF before irradiation effectively improved morphofunctional parameters caused by RILD, restoring histological arrangement, promoting liver regeneration, preserving normal organelles distribution, and glycogen granules. The amount of OV-6 and F4/80-positive cells increased, and α-SMA positive cells’ presence was normalized. Additionally, prior G-CSF administration preserved serum biochemical parameters and increased the survival rates (100%). On the other hand, after irradiation, the treatment showed a slight improvement in survival rates (79%) and did not ameliorate RILD. Overall, our data suggest that G-CSF administration before radiation might be an immunotherapeutic alternative to radiotherapy planning to avoid RILD.

## Introduction

Liver cancer is one of the most common causes of death worldwide (Sia et al., 2017). Currently, liver cancer is understood as a multistep and complex disease resulting from many factors including chronic injury post hepatitis caused by B and C viral infections, liver cirrhosis, exacerbated alcohol consumption, and a high-fat diet (McGlynn et al., 2020). Radiotherapy has been used widely in cancer treatment (Kreidieh et al., 2019). As a result of this common strategy, radiation-induced liver disease (RILD) has emerged such an adverse side effect of liver cancer treatment (Nakajima et al., 2018). RILD can occur as an acute response during the treatment, after a few weeks, or still, as a late response, after months or years of the radiotherapy has ended (Koay et al., 2018). As a result of liver radiotherapy exposition, several effects can occur, including vascular and neural system damage. These effects can promote disorders in the brain via the liver-brain inflammation axis and contributes to inflammatory diseases and alteration on patients’ behavior (D’Mello and Swain, 2011).

Besides elevated hepatic enzymes, RILD can promote extracellular matrix changes and inflammation-related responses leading to liver fibrosis development. Unfortunately, these events worsen the symptoms associated with liver cancer development and contribute to high taxes of mortality (Kim and Jung, 2017). To mitigate the devastating clinical problems associated with RILD, several studies have focussed on establishing radiation protocols to guide patients’ treatment planning (Koay et al., 2018). However, no efficient pharmacological strategies have provided a curative treatment of RILD. Modern radiation techniques and strategies based on the Childs Pugh score and stem cell-based approaches have been used (Pan et al., 2010; Francois et al., 2013; Zhang et al., 2014; Chen et al., 2015; Petrowsky et al., 2020). However, clinical and pre-clinical studies revealed modest, controversial, and transient results (Benderitter et al., 2014). On the other hand, attractive clinical approaches have been used to reach better results.

In this scenario, granulocyte colony-stimulating factor (G-CSF) has arisen as such a potential tool. This glycoprotein has worldwide clinical use, is secreted by the cells of the immune system, fibroblasts, and endothelium, and has been described as a regulator of the proliferation and differentiation of hematopoietic stem/progenitor cells (HSPCs). Also, G-CSF stimulates granulopoiesis, innate immunity, and the differentiation of neural progenitor cells. Several studies have demonstrated that G-CSF treatment induces changes in the blood cell population distribution (Patchen et al., 1990; Gaia et al., 2006; Satyamitra et al., 2017; Wen et al., 2019). G-CSF induces polymorphonuclear and mononuclear myeloid-derived suppressor cells in peripheral blood (Luyckx et al., 2012). A substantial increase of myeloid, erythroid progenitors, and megakaryocytic cells are mobilized after G-CSF treatment (Greenbaum and Link, 2011). Significant induction of hematopoietic stimulation results in the increases of white and red blood cell and platelets values in peripheral blood post-treatment. In some cases, these increased taxes were relatively constant, however, fluctuations are also observed during and after G-CSF treatment. Interestingly, it had been previously shown that G-CSF may induce the generation of low-density granulocytes (Vasconcelos et al., 2006) and monocytes (D’Aveni et al., 2015) in both humans and mice. In addition, Vela-Ojeda et al. (2006) reported that G-CSF was able to mobilize different lymphocytes subsets of T cells, Treg, and dendritic cells to the blood. On the other hand, no difference regarding B, NK, and NKT cells was found in the blood after G-CSF treatment.

Besides, G-CSF contributes to ameliorating inflammation’s pathogenesis and improving the response to infection, decreasing the release of pro-inflammatory cytokines and regulating the release of anti-inflammatory cytokines (Rewerts et al., 1998; Boneberg and Hartung, 2002; Suzuki et al., 2008). The beneficial effects of G-CSF in the treatment of various injuries, including bone fracture healing (Moukoko et al., 2018), thin endometrium and recurrent implantation failure (Kamath et al., 2017), leukemia (Feng et al., 2018), and other diseases such as cardiovascular diseases (D’Amario et al., 2018) have been shown. According to Wang et al. (2020), G-CSF was able to reduce infarction size and interstitial collagen fiber deposition in the myocardium, attenuating myocardial remodeling and ventricular arrhythmia in a rabbit model of coronary microembolization. These studies have demonstrated that the mobilization mechanism is related to its capacity to stimulate cell migration and promote anti-apoptotic and anti-inflammatory properties.

G-CSF has been used in several non-clinical models to promote liver recovery in different experimental models of liver disease. Evidence of positive hepatic effects of G-CSF has been reported. Some studies revealed liver regeneration by mobilizing bone marrow mesenchymal stromal/stem cells (BM-MSCs) to the injured liver after G-CSF administration (Liu et al., 2006; Yanagawa et al., 2019). This mobilization causes changes by circulating stem cells into the peripheral blood. The presence of CD34^+^ positive stem/progenitor cells was detected in the blood after G-CSF treatment in patients with liver disease and was related to liver function improvement in these patients. (Gazitt et al., 2000; Petit et al., 2002; Gordon et al., 2006). On the other hand, some studies showed that G-CSF could act directly on liver tissue ameliorating non-alcoholic hepatic steatosis (Song et al., 2013) and preventing the development of hepatic steatosis in rats (Song et al., 2015).

The association of radiotherapy and immunostimulatory approaches have been explored, and beneficial effects were shown in these studies (Goedegebuure et al., 2019). However, the hepatic benefits against RILD promoted by immunostimulatory G-CSF treatment before and after radiotherapy exposition remain unclear.

We previously showed that a chronic liquid alcohol feeding associated with a hepatotoxic agent such as carbon tetrachloride (CCl_4_) induces liver fibrosis and cirrhosis in rats (Carvalho et al., 2008; Dias et al., 2008; Quintanilha et al., 2008). Based on this experience, in this work, we designed a model of alcohol plus irradiation exposition to generate RILD in C57BL/6 mice. Chronic alcohol administration contributes as an adjuvant to promote previous toxic effects in the liver before irradiation experiments. Ethanol is a small molecule commonly used to promote alcohol-induced liver injury. Several studies, using both human and animal models, have reported that ethanol promotes key triggers such as cell injury, inflammation, oxidative stress, and steatosis in the liver (Mathews et al., 2014; Rocco et al., 2014; Louvet and Mathurin, 2015). In addition, the chronic cytotoxic effects of ethanol metabolism are also responsible for apoptosis induction. The long-term ethanol administration used in this study makes hepatocytes more sensitive to oxidative stress, amplifying the effects after irradiation exposure promoting RILD outcomes in C57BL/6 mice.

Furthermore, the efficiency of G-CSF treatment of RILD has been reported (Li et al., 2010). However, the effect of G-CSF in liver regeneration after radiation-induced liver disease in association with a previous alcohol-induced liver injury is unknown. Based on these studies in literature, we hypothesized that immunotherapy with G-CSF could be protective in a mouse model of RILD.

To address this issue, in this study, we evaluated the effects of G-CSF treatment before and after radiotherapy (18 Gy) exposure in association with a previous alcohol-induced liver injury in C57BL/6 mice.

## Materials and Methods

### Animals

Experiments were performed in 12-week-old female and male C57BL/6 mice (*n* = 120), weighing 18–20 g. All procedures were performed in conformity with the Guide for the Care and Use of Laboratory Animals (DHHS Publication No. (NIH) 85-23, revised 1996, Office of Science and Health Reports, Bethesda, MD 20892) and were approved by the Ethics Committee for Animal Use of the Federal University of Rio de Janeiro under the number 162/13. The mice were obtained from Carlos Chagas Filho Biophysics Institute (IBCCF—Rio de Janeiro/Brazil) and housed at a controlled temperature (23°C) with a 12:12-h light-dark cycle and free access to standard mice chow.

### Experimental Design

#### Radiation-Induced Liver Disease and G-CSF Treatment

The animal’s livers were exposed to a single dose of radiation (18 Gy) in a linear accelerator Clinac 2100 CD (Varian Inc., United States) used for radiotherapy. Measurements were performed using a photon beam of nominal energy of 6 MV and a dose rate of 240 Gy/min. Firstly, under anesthesia with 1.5% isoflurane gas at a flow rate of 1 L/min of oxygen, the animals were immobilized in the supine position. Then, a 5 × 2 cm (width x height) field below the xiphoid process was focally irradiated. To ensure that 100% of the dose would be irradiated directly into the liver, a 1 cm bolus was used in the linear accelerator. During three consecutive days, at 10 am, pre-or post-irradiation mice received recombinant human G-CSF (100 μg/kg/day of Filgrastine ^®^, Blaú Farmacêutica SA, Brazil), *via* subcutaneous (sc) in 200 µl of glycosylated solution (5%) (Werneck-de-Castro et al., 2006; Song et al., 2013). All irradiated groups were submitted to a previous alcohol-induced liver injury protocol with filtered and autoclaved water containing 5% alcohol (Caninha 51, Companhia Muller, Brazil) for 90 days.

#### Experimental Groups

C57BL/6 mice (*n* = 120) were randomly assigned to one of the four following treatment groups: Control (CTRL, *n* = 30); alcohol, irradiation and glucose solution 5% (iNon-treated, *n* = 30); irradiation, alcohol and G-CSF pre-irradiation (iGCSF-pre, *n* = 30); and irradiation, alcohol and G-CSF post-irradiation (iGCSF-post, *n* = 30). All parameters were analyzed at 7, 30 and 60 days post-irradiation (dpir).

### Serum Biochemical Analysis

After euthanasia, 200 µl of blood was collected by cardiac puncture. Then, the blood was centrifuged at 4,000 ×g for 10 min to separate the serum. Albumin measurements (Green Bromocresol method, Labtest ^®^, Brazil, cat. 19) and Alanine aminotransferase (ALT) enzyme (UV-IFCC, Labtest ^®^, Brazil, cat. 108) were carried out in the Bio 200F^®^ semi-automatic device (Bio Plus, Brazil). Samples from 10 animals was used in each analyzed group.

### Ultrasound Analysis

The animals were anesthetized with 1.5% isoflurane gas at a flow rate of 1 L/min of oxygen. They were trichotomized in the pericordial region and examined in the supine position with the Vevo 770 device (Visual Sonics, Canada), coupled to a 30 MHz transducer. The liver and kidney were evaluated by multiple transversal and longitudinal scans. The liver aspects and echogenicity were observed beyond the echographic relationship between the liver and the renal cortex.

### Computed Tomography—Full Body Scanning

The CT of an Optima 560 PET/CT scanner (GE Healthcare, United States) was used. The X-ray tube was adjusted to 140 kV and 250–300 mAs. The total time for each scan was 12 s. The images were taken 48 h after the intravenous administration of 100 µl of EXITRON NANO 12000 (Miltenyi Biotec, United States, cat. 130-095-698). The analyzes were performed using the program Osirix ^®^ Lite (Pixmeo, Switzerland). The technique consisted of assessing the degree of hepatic steatosis, using the attenuation values found in the liver (F) and spleen (B). In the evaluation, three regions of interest (ROI) with an area of 3.02 mm^2^ were selected, both in the liver and the spleen, and from the average of the values found, a ratio was found F/(B). The results obtained can be distributed into three categories of fat infiltration, being mild (0.7 <F/(B) < 1.0), moderate (0.5 <F/(B) ≤ 0.7) and severe (F/(B) ≤ 0.5). The calculation of liver volume was performed using the compute volume tool after segmentation of the organ.

### Magnetic Resonance Image

The animals were anesthetized with 1.5% isoflurane gas at a flow rate of 1 L/min of oxygen. The mice were positioned in the supine position. Then images were acquired using a magnetic resonance imaging (MRI) 7.0 T 210 Bore Actively Screened Refrigerated Magnet System (Varian Inc., United States). Body temperature was maintained by a hot air system coupled to the device. The T1 weighted spin-echo pulse sequences were acquired pre and post gadolinium intraperitoneal injection (contrast agent: Dotarem^®^, 0.2 ml/kg). (Repetition Time (TR) = 500 ms, Time to Echo (TE) = 18 ms, 20 slices, without gap, Thickness = 1 mm, matrix = 128 × 128, Field of View (FOV) = 3 × 3 cm^2^, averages = 12, acquisition time = 12 min).

### Processing Liver Tissue Samples

After liver function analysis was performed, the animals received intraperitoneal heparin injection (50 μl—Hepamax -S^®^ 5,000 IU/ml) and were euthanized by anesthetic overdose (Ketamine Hydrochloride 10%—240 mg/kg and Xylazine Hydrochloride 2%—60 mg/kg intraperitoneal). Then the livers were surgically removed and perfused with Phosphate Buffer Saline (PBS) 1× for subsequent morphological analysis.

#### Hematoxylin and Eosin Staining and Silver Impregnation

The liver tissue was fixed in formalin 10% (formaldehyde powder 95%, Sigma-Aldrich, EUA, cat. 158127 and distilled water—9:1), dehydrated in an increasing series of ethyl alcohol (VETEC, Brazil, cat. V107), diaphonized in xylol (VETEC, Brazil, cat. V142) and impregnated with paraffin (Easypath, Brazil, cat. EP-21-20068). Four μm tissue sections were stained with Hematoxylin (VETEC, Brazil, cat. V630) and Eosin (Sigma, Japan, cat. E4382) as worldwide described or impregnated with silver using the Reticulin kit (Easypath, Brazil, cat. EP-12-20021). Liver slices were scanned on the Pannoramic MIDI device (3D Histech, Hungary). A qualitative analysis was carried out, in which 20 fields were evaluated per slice of each animal, with a 40× objective.

#### Immunofluorescence

The liver tissue was fixed in 4% paraformaldehyde, immersed in increasing gradients of D-sucrose (Sigma-Aldrich, EUA, cat. 228), and impregnated with a water-soluble resin (OCT—TissueTek ^®^, Sakura, United States, cat. 4583). Sections (5 μM) of tissue in coverslips were incubated for 30 min at room temperature with 5% IgG-free bovine serum albumin (BSA) (Sigma-Aldrich, United States, cat. A6003) to reduce nonspecific binding. This was followed by incubation with primary antibodies to detect Alpha Smooth Muscle Actin (α- SMA, Abcam, United States, cat. Ab7817) (dilution 1:400), F4/80 macrophages (Abcam, United States, cat. Ab6640) (dilution 1:200), Cytokeratin 18 (CK18, Abcam, United States, cat. Ab668-100) (dilution 1:200), Cytokeratin 19 (CK19, Abcam, United States, cat. Ab15463) (dilution 1:50), Alpha Fetoprotein (AFP, Abcam, United States, cat. Ab46799) (dilution 1:250), and Oval Cells (Ov6, Abcam, United States, cat. Ab2020) (dilution 1:400) for 12 h at −4°C temperature. After, the coverslips were then washed three times with PBS and incubated for 2 h at room temperature with secondary antibodies Alexa Fluor 488 (Life Technologies, United States, cat. A1134) (dilution 1:300) and Alexa Fluor 647 (Life Technologies, United States, cat. A21245) (dilution 1:300).

The slices in coverslips were then washed three times with PBS and mounted with an anti-fading solution (ProLong™ Gold Antifade Mounted with DAPI—Life Technologies, United States, cat. P36931). The nuclei of the cells in tissues were labeled with intercalating nuclear solution DAPI (4,6-diamidino-2-phenylindole) associated with the anti-fading solution. Confocal images were obtained using an LSM 710 Quasar Zeiss confocal microscope (Carl Zeiss, Oberkochen, Germany). To quantify the expression of α-SMA, F4/80, OV6, and CK18, three images (each) were captured and analyzed using the Fiji software (ImageJ) with the “Process Make binary tool”. The results represent the sign value in percentage of the area occupied in relation to the total area of the image in pixels (512 × 512).

#### Transmission Electron Microscopy

Liver samples were fixed in 2.5% glutaraldehyde (Electron Microscopy Sciences, United States, cat. 16220), post-fixed with osmium tetroxide (Electron Microscopy Sciences, United States, cat. 19100) and potassium ferrocyanide (Electron Microscopy Sciences, United States, cat. 20150) for 60 min, dehydrated with increasing series of PA acetone (VETEC, BRAZIL, cat. 187) and infiltrated with EPOXI resin (EMbed812, Electron Microscopy Sciences, United States, cat. 14120). After polymerization, 1 µm semi-fine slices were made to choose the region to be analyzed. Then, 70 nm ultrathin sections were made; these were contrasted with 1% uranyl acetate (Electron Microscopy Sciences, United States, cat. 22400) and 1% lead citrate (Electron Microscopy Sciences, United States, cat. 17800), and visualized using Jeol transmission electron microscope JEM-1011 (JEOL Ltd., Akishima, Japan). Digital micrographs were captured using an ORIUS CCD digital camera (Gatan Inc., United States) at 5,000× magnification, and later a qualitative analysis was carried out. The mitochondria and endoplasmic reticulum integrity, amount of glycogen grains, and cytoplasm were evaluated in 15 fields per grid of each animal. (*n* = 5/group).

### Statistical Analysis

One-way or two-way analysis of variance (ANOVA) with Tukey posttest was used. Survival data were analyzed with the log-rank (Mantel–Cox) test. The data were presented as mean ± standard error of the mean in all tests, the minimum degree of significance considered was 95% (*p* < 0.05), and statistical calculations were performed using the Graph Pad Prism ™ 9 program.

## Results

### G-CSF Protected the Liver Against Damage Promoted by Radiation-Induced Liver Injury

**FIGURE 1:**
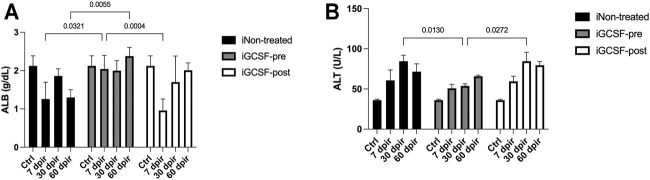
Blood markers of liver function and injury before and after G-CSF treatment. Serum albumin (ALB) levels measured on experimental days 7, 30, and 60 in iNon-treated Group, iGCSF-pre and, iGCSF-post group **(A)**. Serum alanine aminotransferase (ALT) levels measured on experimental days 7, 30 and, 60 in iNon-treated Group, iGCSF-pre and, iGCSF-post group **(B)**. Multiple comparisons using two-way analysis of variance (ANOVA) followed by Tukey’s test were used. Data are represented as means ± SEM, *p* < 0.05 was considered significant, where *p < 0.05; **p < 0.01 and ***p < 0.001) (*n* = 10 per Group).

While a significant decrease in serum ALB values at 7 dpir and 60 dpir (CTRL 2.207 ± 0.04, 7 dpir 1.205 ± 0.09, 60 dpir 1.298 ± 0.09 g/dl) was detected in the untreated group (iNon-treated), the treatment with G-CSF before irradiation (iGCSF-pre) was significantly able to prevent the decrease in serum ALB values, remaining similar to the control at 7 dpir (*p* = 0.0321) and 60 dpir (*p* = 0.0055) (CTRL 2.207 ± 0.04, 7 dpir 2.040 ± 0.12, 60 dpir 2.380 ± 0.08 g/dl). On other hand, in the group that received treatment after radiotherapy (iGCSF-post Group), we observed that serum ALB values decreased (*p* = 0.0004) at 7 dpir but they started to increase again after 30 dpir (CTRL 2.207 ± 0.04, 7 dpir 1.003 ± 0.09, 30 dpir 1.701 ± 0.25, 60 dpir 2.009 ± 0.06 g/dl). (Figure 1A).

To identify liver injury in irradiated mice, we evaluated serum ALT levels. The iNon-treated Group (*p* = 0.0130) (CTRL 36.00 ± 1.17, 30 dpir 84.58 ± 7.31 U/L) and iGCSF-post Group (CTRL 36.00 ± 1.17, 30 dpir 84.50 ± 11.24 U/L) showed significantly elevated levels of ALT at 30 dpir in comparison with iGCSF-pre-Group (*p* = 0.0272) (CTRL 36.00 ± 1.17, 30 dpir 53.80 ± 2.60 U/L) (Figure 1B).

**FIGURE 2:**
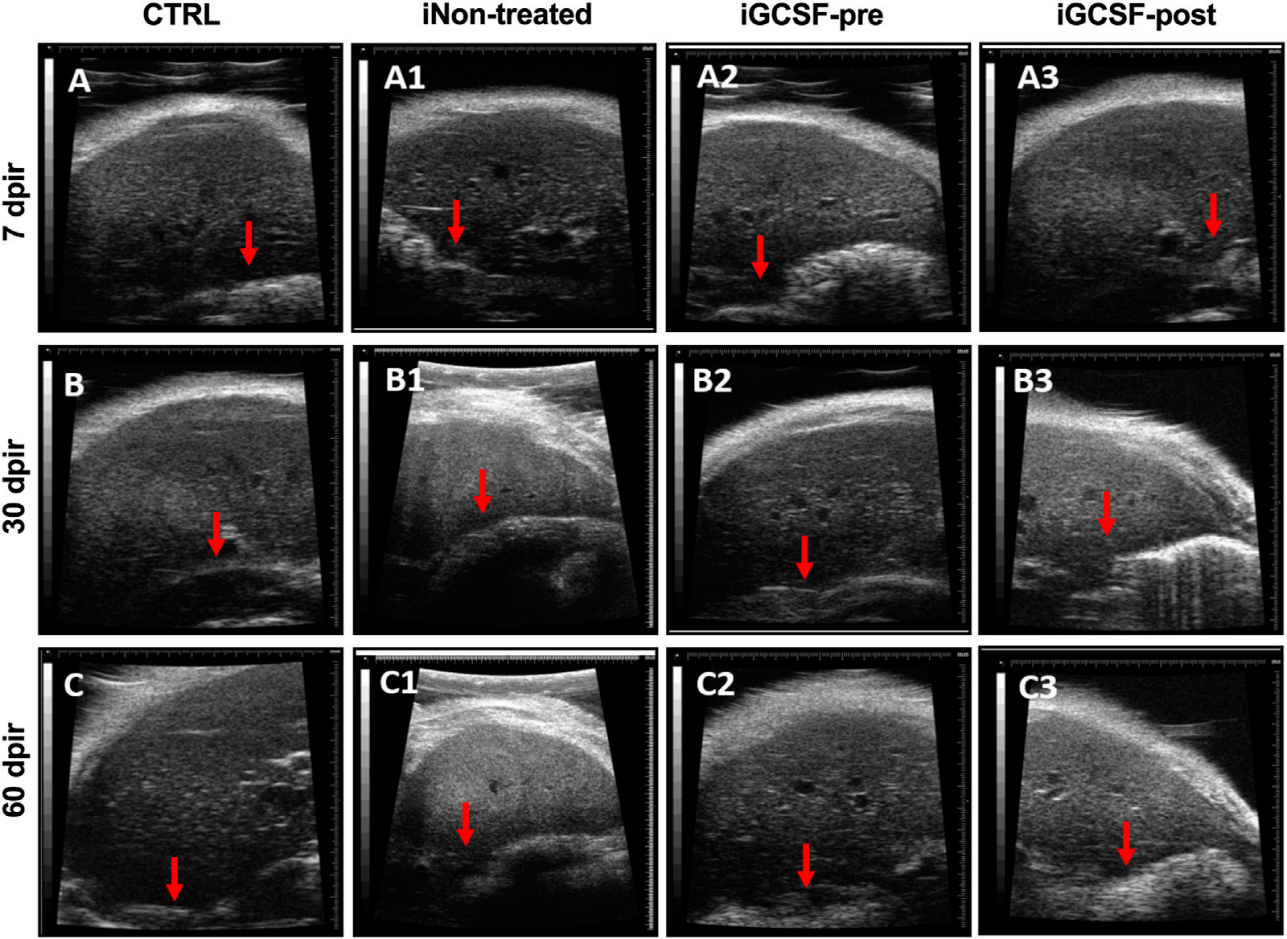
Liver ultrasound findings. Ultrasound images obtained from CTRL **(A–C)**, iNon-treated **(A1–C1)**, iGCSF-pre **(A2–C2)** and, iGCSF-post group **(A3–C3)** at 7, 30- and 60-days post-irradiation. Images obtained from a healthy animal show normal liver echogenicity and a homogeneous, smooth and, regular surface (indicated by red arrow) **(A–C)**. iNon-treated group images **(A1–C1)** showed liver parenchyma with finely and slightly heterogeneous and increased echogenicity. Besides, a lobulated liver surface was too observed (indicated by red arrow). Similarities were observed in animals of the iGCSF-pre Group in 30 **(B2)** and 60 dpir **(C2)** and all animals in the iGCSF-post Group **(A3–C3)**. Images obtained from iGCSF-pre in 7 dpir **(A2)** revealed similarities with CTRL group, maintaining its homogeneous echogenicity and regular surface (*n* = 8 per Group).

After performing serum biochemical analysis, we investigated the liver parenchyma’s appearance and echogenicity by high-resolution ultrasound. After qualitative analysis, we observed that G-CSF treatment prevented changes in the liver parenchyma up to 7 dpir (Figure 2A2), maintaining its homogeneous echogenicity and regular surface as similar to that found in the control group (Figures 2A–C). However, after 30 dpir, in this group (Figures 2A2–C2) and the iGCSF-post Group (Figures 2A1–C1), the liver parenchyma showed a heterogeneous and slightly increased echogenicity. Besides, a lobulated hepatic surface, as found in the untreated group (iNon-treated), was identified (Figures 2A1–C1). After incidence analysis, we observed that all animals (*n* = 8) had a liver with a homogeneous and regular surface in the CTRL Group, while all animals (*n* = 8) showed heterogeneous and slightly increased echogenicity in the iNon-treated Group. Regarding the iGCSF-pre Group, all animals showed smooth and homogeneous surfaces up to 7 dpi and two animals showed heterogeneous and slightly increased echogenicity after 30 dpir. In the iGCSF-post Group, we identified six animals with a lobulated liver surface and one animal with heterogeneous and slightly increased echogenicity.

**FIGURE 3:**
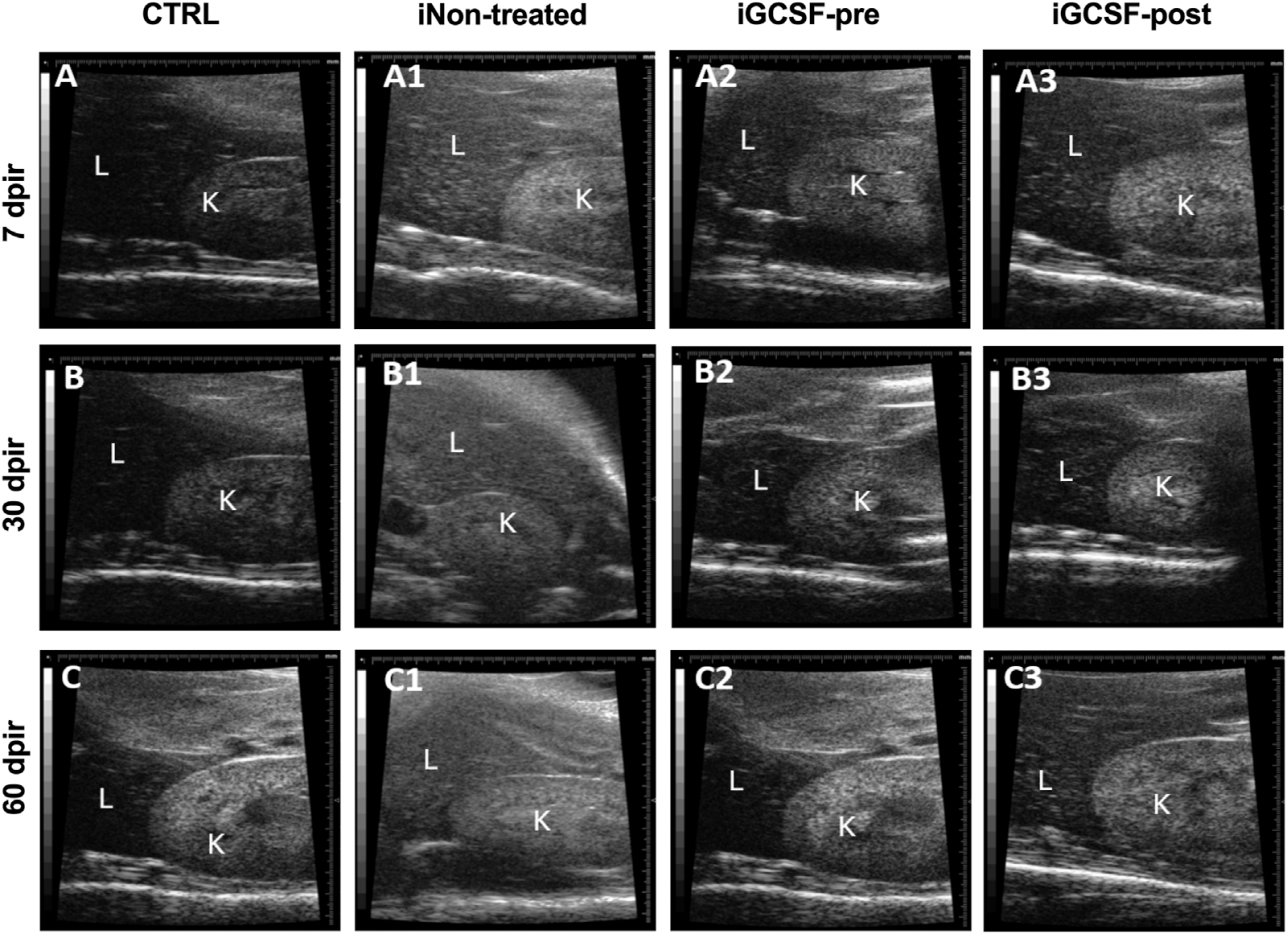
Hepatorenal ultrasound findings. Ultrasound images obtained from CTRL **(A–C)**, iNon-treated **(A1–C1)**, iGCSF-pre **(A2–C2)** and, iGCSF-post group **(A3–C3)** at 7, 30- and 60-day post-irradiation. Images obtained from CTRL group **(A–C)** and iGCSF-pre Group **(A2–C2)** showed that the liver (L) was less echogenic than the kidney (K). Echogenicity similarities between liver and kidney were observed in 7 dpir **(A1)**. Changes in hepatorenal echogenicity were observed in 60 dpir in iGCSF-post, where the liver was hyperechogenic concerning the renal parenchyma (*n* = 8 per Group).

Furthermore, a comparison between the echogenic pattern of the hepatic and renal parenchyma was performed. We observed that in the iGCSF-pre-Group (Figures 3A2–C2), the liver parenchyma was hypoechogenic in relation to the renal parenchyma, as well as the CTRL Group (Figures 3A,C), while in the iNon-treated Group, a kidney isoechogenic hepatic parenchyma was observed at 7 and 30 dpir (Figures 3A1–B1). At 60 dpir we observed that the liver was hyperechogenic in relation to the renal parenchyma (Figure 3C1). In the iGCSF-post Group (Figures 3A3–C3) the hepatic parenchyma echogenicity was lower than the renal parenchyma but with a notable tendency to increase at 60 dpir (Figure 3C3).

**FIGURE 4:**
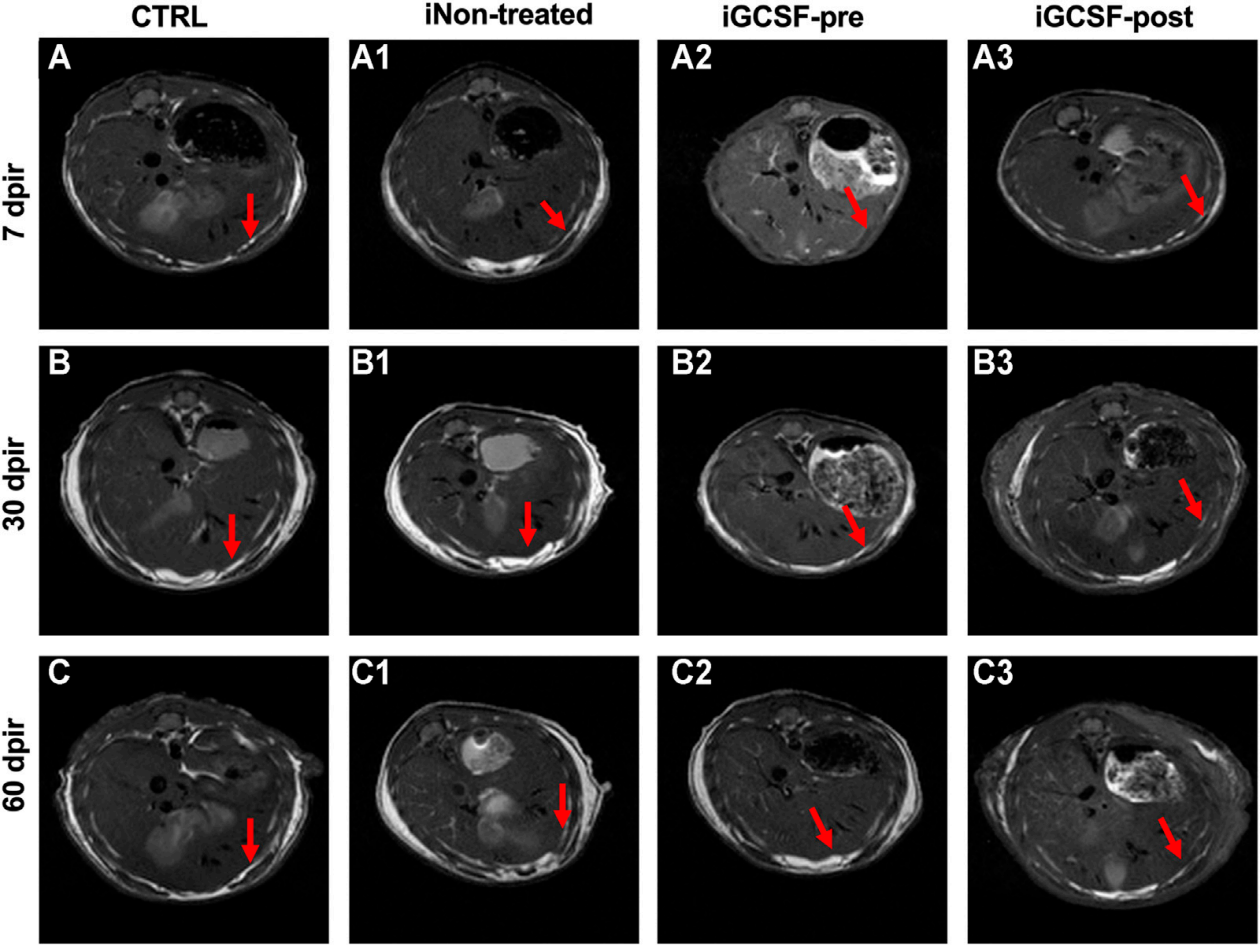
Magnetic resonance image findings. MRI images obtained from CTRL **(A–C)**, iNon-treated **(A1–C1)**, iGCSF-pre **(A2–C2)** and, iGCSF-post Group **(A3–C3)** at 7, 30- and 60-day post-irradiation. Images obtained from CTRL Group **(A–C)** revealed liver parenchyma with diffusely homogeneous signal intensity and regular contours (indicated by arrows). Irregular contours (indicated by arrows) were observed in iNon-treated **(A1–C1)** and, iGCSF-post Groups. In the iGCSF-pre Group, MR images suggest that the signal intensity was lower than in iNon-treated and iGCSF-post Groups, but it also showed irregular contours (indicated by arrows) **(Panels A2–C2)**. All images were acquired before gadolinium intraperitoneal injection (*n* = 10 per Group).

In addition to high-resolution ultrasound analysis, the liver surface was also observed by magnetic resonance image (MRI) pre- and post-gadolinium-based contrast agent. In the CTRL Group, MRI analysis showed a smooth liver surface in all analyzed animals (Figures 4A–C). In the iNon-treated Groups, all the animals showed the contour of a lobulated liver (Figures 4A1–C1) while in the iGCSF-post Group (Figures 4A3–C3) the liver contour was nodular or irregular (eight animals had a smooth surface, and 2 animals were slightly lobulated). However, in the iGCSF-pre-Group (Figures 4A2–C2), the liver contour was slightly lobulated, and sometimes even smooth (six animals had a smooth surface, and four animals were slightly lobulated).

### Prior Treatment With G-CSF Protect Against Fat Accumulation Into Hepatocytes

**FIGURE 5:**
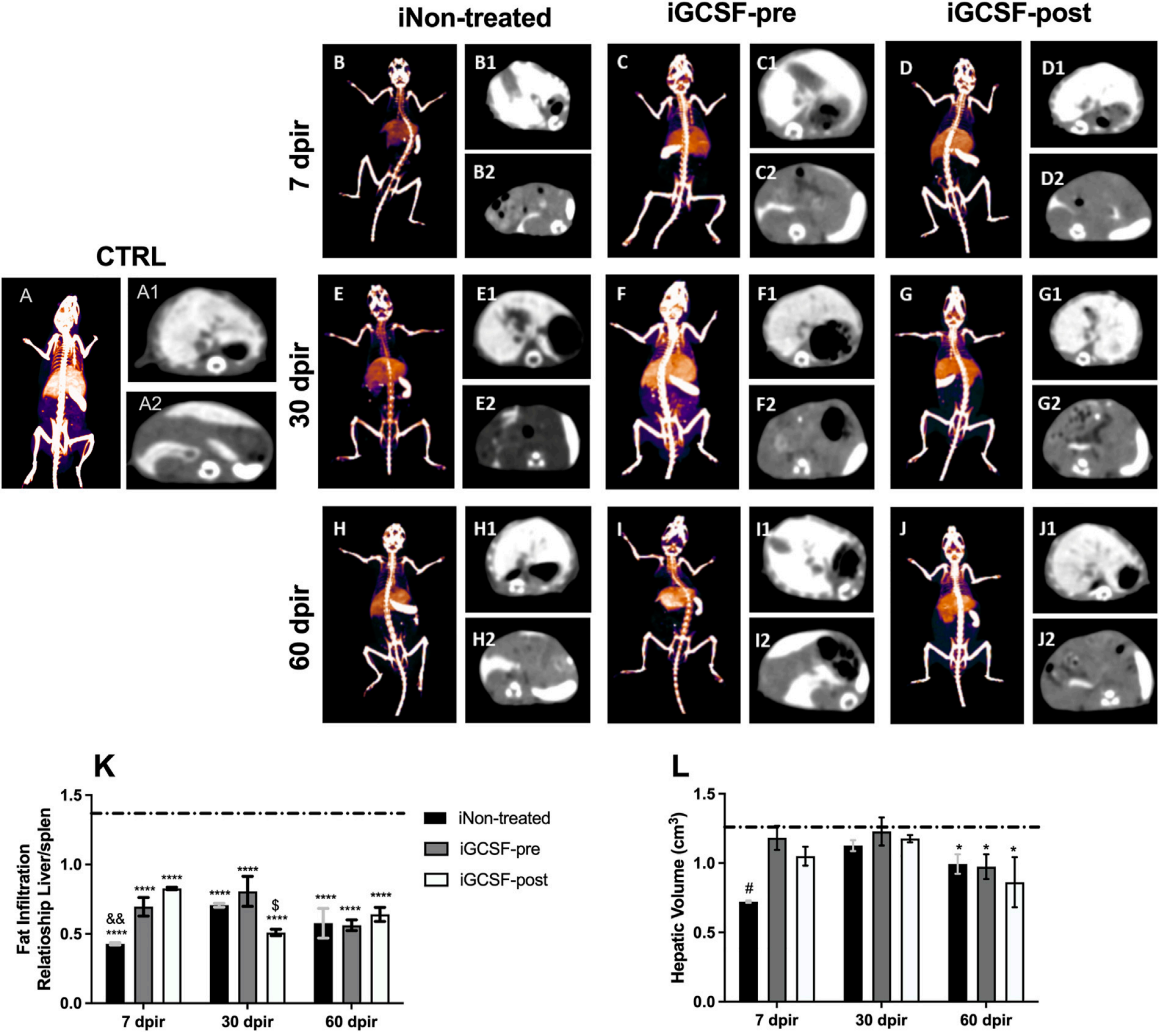
Assessment of liver volume and fat infiltration by computed tomography. Computed tomography 48 h after intravenous administration of EXITRON NANO 12000 contrast. CTRL-3D volumetric reconstruction **(A)**, axial slice showing contrast enhancement by the Liver **(A1)** and Spleen **(A2)** at a control animal. iNon-treated-3D volumetric reconstruction 7 **(B)**, 30 **(E)**, and 60 **(H)** dpir, axial slice showing contrast enhancement by the Liver and Spleen 7, 30 and 60 dpir **(B1,B2, E1,E2, H1,H2)**. iGCSF-pre—3D volumetric reconstruction 7 **(C)**, 30 **(F)** and 60 **(I)** dpir, axial slice showing contrast enhancement by the Liver and Spleen 7, 30 and 60 dpir **(C1,C2, F1,F2, I1,I2)**. iGCSF-post—3D volumetric reconstruction 7 **(D)**, 30 **(G)** and 60 **(J)** dpir, axial slice showing contrast enhancement by the Liver and Spleen 7, 30 and 60 dpir **(D1,D2, G1,G2, J1,J2)**. **(K)** Liver/Spleen ratio among the three groups showing that at 7 dpir iNon-treated group was classified as having severe infiltration, while the groups treated with G-CSF had mild steatosis. At 30 dpir, the iNon-treated Group showed moderate fatty infiltration, the iNon-treated + iGCSF-pre-group light infiltration, and the iNon-treated + iGCSF-post Group had severe steatosis. At 60 dpir, all groups were classified as moderate fatty liver infiltration. **(L)** There is a volumetric reduction of the liver in the iNon-treated and iGCSF-post groups in relation to the control group (dotted line) in all evaluation stages. Multiple comparisons using one-way analysis of variance (ANOVA) were used. Data are represented as means ± SEM (*n* = 5 per Group). *p* < 0.05 was considered significant, where *p < 0.05; ****p < 0.0001.

To investigate the possible hepatic volume alterations, computed tomography analysis was performed (Figure 5). When compared to CTRL Group (Figure 5A), no significant difference was found in the iGCSF-pre Group at 7 dpir (Figure 5C) and 30 dpir (Figure 5F). However, a hepatic volumetric reduction was detected at 60 dpir in the iGCSF-pre Group (*p* = 0.0021) (Figure 5I). Also, computed tomography analysis revealed a volumetric reduction of the liver in the iNon-treated and iGCSF-post Groups in all evaluation stages of evaluation (7, 30, and 60 dpir) (Figures 5D,G,J). However, the most remarkable hepatic volumetric reduction was detected in the iNon-treated Group in 7 dpir (*p* = 0.0127) (Figure 5K).

Besides the hepatic volume assessment, the fatty liver infiltration was also investigated by computed tomography analysis. The assessment of fatty infiltration was performed using a ratio of liver density to that of the spleen, translated into Hounsfield units (HU), which can be classified as mild, moderate, or severe, according to the obtained values. The results indicate that, in the CTRL Group, the liver parenchyma was slightly hyperdense in relation to the spleen, while in all other groups that were exposed to radiation, the liver parenchyma was hypodense in relation to the spleen. On the other hand, the iNon-treated Group was classified as severe liver infiltration after 7 dpir (*p* = 0.0070). Besides, we observed that the treatment with G-CSF was able to prevent or reverse the liver infiltration of fat at 7 dpir, being classified as mild steatosis; however, only the iGCSF-pre-Group maintained this protection at 30 dpir (*p* = 0.0395), when the iGCSF-post Group already had severe infiltration. At 60 dpir, a moderately fatty liver infiltration was observed in all groups (Figure 5L).

### Prior Treatment With G-CSF Improves Liver Histology

**FIGURE 6:**
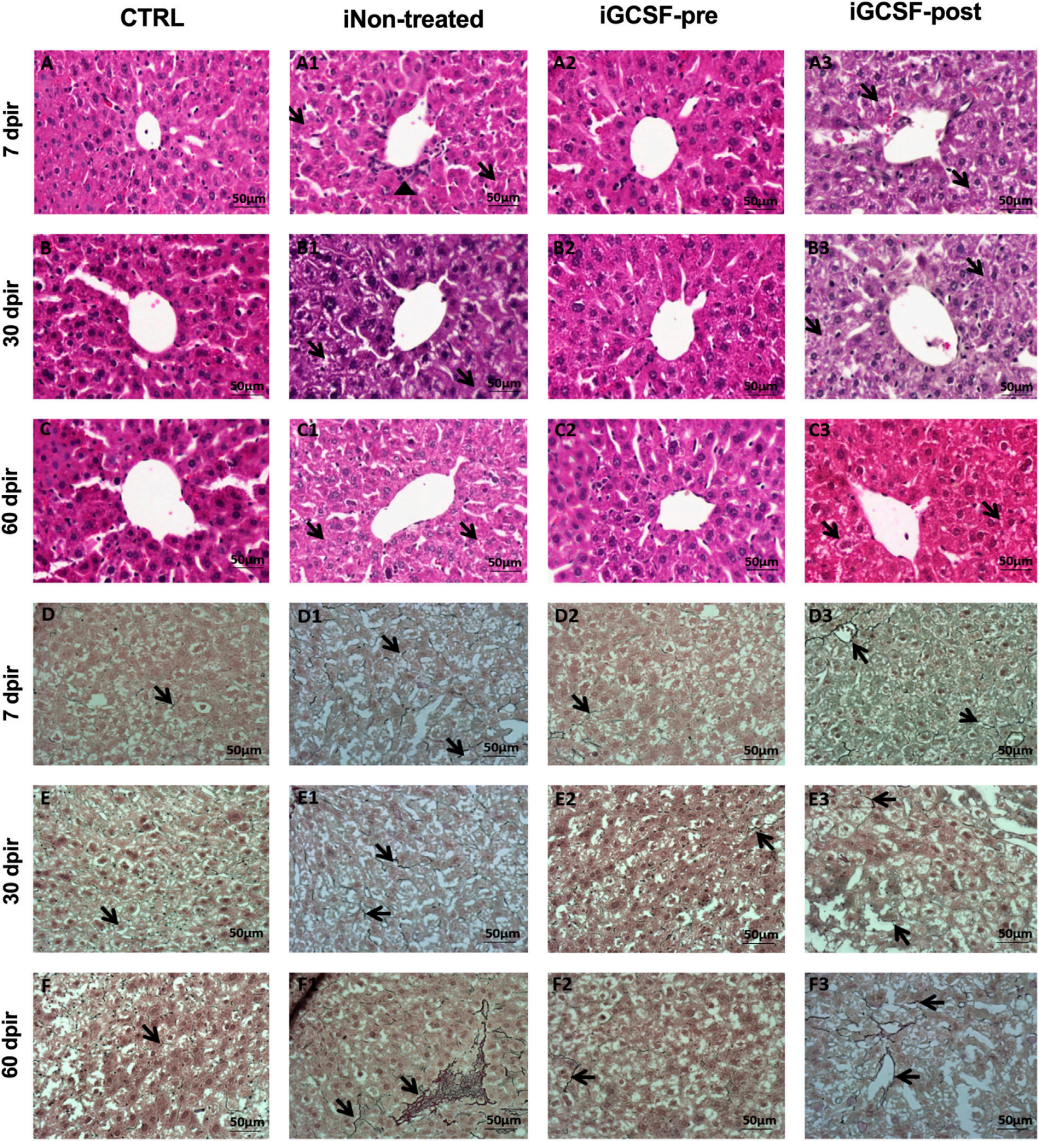
Histopathological assessment of liver tissue. Representative images stained with hematoxylin and eosin (H&E) and reticulin of CTRL **(A–C; D–F)**, iNon-treated **(A1–C1,D1–F1)**, iGCSF-pre **(A2–C2,D2–F2)** and iGCSF-post group (A3-C3; D3-F3) at 7, 30- and 60-day post-irradiation. CTRL Group **(A–C)** H&E-stained sections revealed liver parenchyma with a radial distribution of hepatocytes around the vessels and homogeneous distribution of cytoplasm. In the iNon-treated Group, in 7 dpir **(A1)** the loss of the radial distribution of hepatocytes, beginning cytoplasm vacuolization (indicated by arrows) and polymorphonuclear infiltrate (indicated by arrowhead) was observed. At 30 dpir **(B1)**, most hepatocytes had vacuolization of the cytoplasm and did not show radial distribution around the vessel. At 60 dpir **(C1)** radial distribution was observed, but hepatocytes showed vacuolated cytoplasm (indicated by arrows). A preserved liver parenchyma with a radial distribution of hepatocytes and absence of cytoplasmic vacuolization (indicated by arrows) of hepatocytes were observed in iGCSF-pre **(A2–C2)** group at 7, 30 and, 60 dpir. In the iGCSF-post **(A3–C3)** group, hepatocytes showed vacuolated cytoplasm (arrows) and loss of radial distribution around the vessel at all times analyzed. (*n* = 8 per group). In liver tissue impregnated with silver (Reticulin) histological sections, the CTRL group **(D–F)** showed the presence of few reticulin fibers (indicated by arrows). iNon-treated group images **(D1–F1)** showed an increase in these reticulin fibers (arrows) from 7 dpir **(D1)** to 60 dpir **(F1)**. In the iGCSF-pre Group **(D2–F2)** it was observed that the tissue had fewer reticular fibers (arrows) than the iNon-treated Group, but more fibers (arrows) were found than in the CTRL group. In the iGCSF-post Group **(D–D2)**, there was also an increase in fibers (indicated by arrows) concerning CTRL (*n* = 8 per Group). Scale bars: 50 µm.

To investigate the histological benefits of G-CSF treatment in our liver-induced injury model, histopathological analysis was performed. The iNon-treated Group (all analyzed animals) H&E-stained sections showed the loss of hepatocytes radial distribution, including some hepatocytes at the beginning of cytoplasm vacuolization and polymorphonuclear cells infiltrate around the vessels (Figure 6A1) at 7 dpir. In contrast, most hepatocytes presented vacuolization of the cytoplasm and did not present a radial distribution around the vessels at 30 dpir (Figure 6B1). However, cytoplasm vacuolization remained, and radial hepatocyte distribution was observed at 60 dpir (Figure 6C1). On other hand, similar to the CTRL Group (Figures 6A–C), a preserved liver parenchyma with a radial distribution of hepatocytes, homogeneous distribution of cytoplasm within cells, and an absence of cytoplasmic vacuolization were observed in the iGCSF-pre-Group at all analyzed times (7, 30, and 60 dpir) (Figures 6C–C2). Seven animals had preserved liver parenchyma with a radial distribution of hepatocytes and an absence of cytoplasmic vacuolization in the iGCSF-pre-Group. In the iGCSF-post Group (Figures 6A3–C3), at all analyzed times, we observed a vacuolated cytoplasm and the parenchyma presented a loss of radial distribution of hepatocytes around the vessels in all analyzed animals.

After performing H&E staining analysis, reticulin staining was also performed. Histological assessment of the fibers revealed that the iGCSF-pre-Group (Figures 6D2–F2) presented more reticulin fibers than the CTRL Group (Figures 6D–F), but less than the iNon-treated Group (Figures 6D1–F2 and iGCSF-post Group (Figures 6D3–F3). Incidence analysis revealed that five animals had an average amount of reticular fibers in the liver parenchyma, while three animals had fewer reticular fibers in the iGCSF-pre-Group. In addition, the increment of reticular fibers was more observable in the iGCSF-post Group (all analyzed animals) from 7 dpir to 60 dpir.

### Prior Treatment With G-CSF Contributes to Liver Tissue Ultrastructure Preservation

**FIGURE 7:**
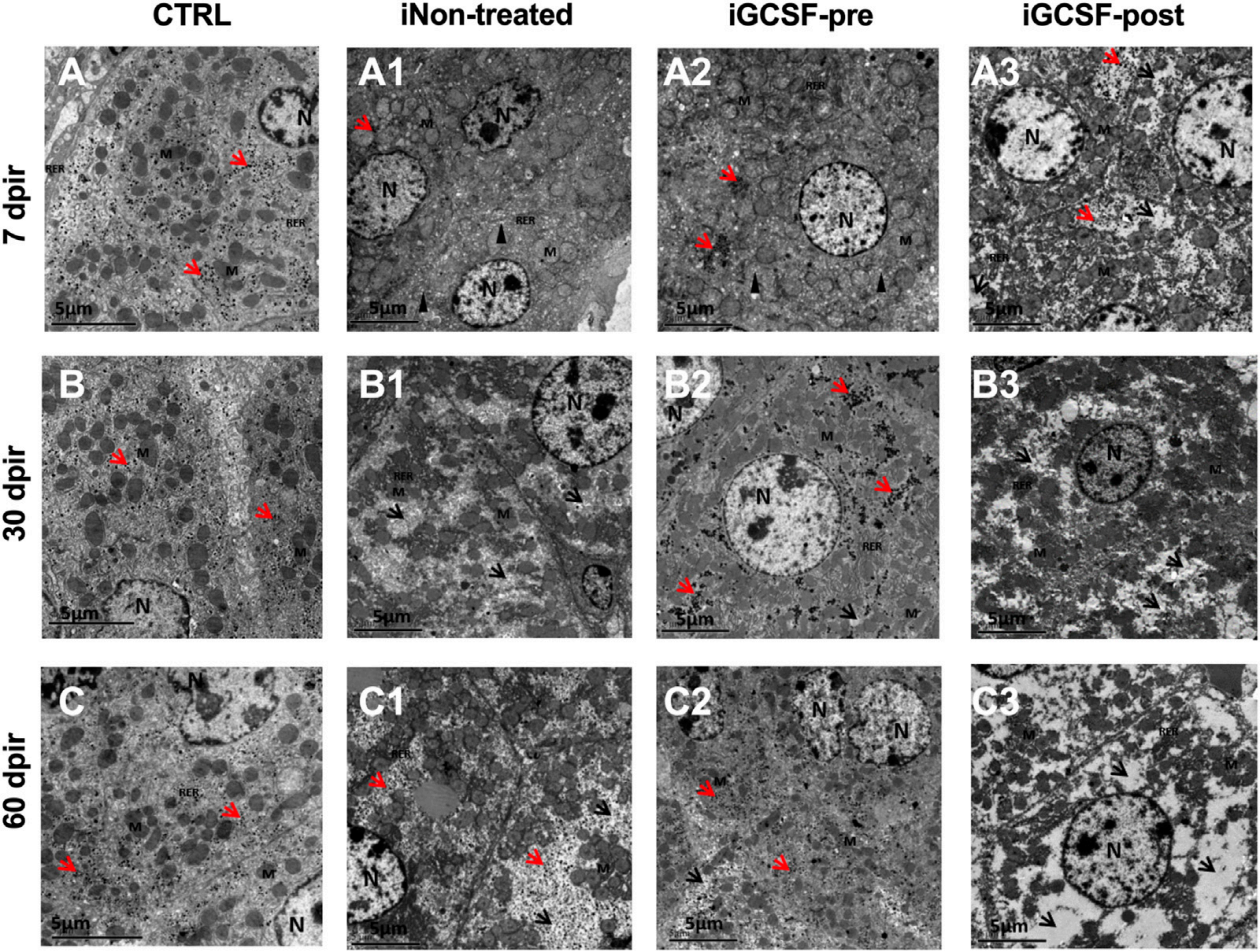
Electron micrographs representative of transmission electron microscopy analysis (TEM). TEM images of CTRL group **(A–C)** showed well-condensed cytoplasm with several organelles, well-preserved mitochondria, rough endoplasmic reticulum, and distributed glycogen granules. iNon-treated Group **(A1–C1)** in 7 dpir **(A1)** showed more evident smooth endoplasmic reticulum (SER) cisterns, ruptured mitochondria, and loss of ridges, in addition to the rarer cytoplasm. At 30 **(B1)** and 60 **(C1)** dpir, a rarer cytoplasm was observed with an absence of organelles, mitochondria with rupture of the outer membrane, absence of mitochondrial ridges, and reduction of glycogen granules. In the iGCSF-pre **(A2–C2)** group, with 7 dpir **(A2)**, well-condensed cytoplasm was observed with preserved mitochondria, RER, glycogen granules, similar to the CTRL group, but with more evident SER cisterns. With 30 **(B2)** and 60 dpir **(C2)**, a large amount of glycogen granules, reduction of the cisterns of SER, few regions of rarefied cytoplasm and well-preserved mitochondria were observed. In the iGCSF-post Group **(A3–C3)**, at all times analyzed, rarefied cytoplasm, degraded, ruptured, and crested mitochondria, fragmented RER, and presence of glycogen granules were only observed in 7 dpir **(A3)**. N, nuclei; M, Mitochondria; RER, rough endoplasmic reticulum; SER: smooth endoplasmic reticulum; Arrowhead, dilated smooth endoplasmic reticulum cistern; Red arrow, glycogen granules; Black arrowhead, rarefied cytoplasm. (*n* = 5 per group). Scale bars: 5 µm.

The ultrastructural analysis confirmed similarities between the iGCSF-pre Group and the CTRL Group (Figures 7A–C) and revealed a well-condensed cytoplasm with normal organelles distribution and mitochondria, rough endoplasmic reticulum, and preserved glycogen granules in the iGCSF-pre Group at 7 dpir (Figure 7A2). Also, an evident cisternal smooth endoplasmic reticulum was observed. It is essential to mention that a large amount of glycogen granules, reduced smooth endoplasmic reticulum cisterns, few regions of the thin cytoplasm, and preserved mitochondria were observed, demonstrating that organ repair process after lesion at 30 and 60 dpir (Figures 7B2,C2). These findings were observed in four of five analyzed animals in the iGCSF-pre-Group.

In the treated group after radiotherapy (iGCSF-post) (Figures 7A3–C3), a rarefied cytoplasm with degraded, ruptured, and crested mitochondria and fragmented endoplasmic reticulum were observed at all analyzed times in all analyzed animals. Besides, glycogen granules were identified only at 7 dpir (Figure 7A3) and were absent at all other analyzed times (30 dpir (Figure 7B3) and 60 dpir (Figure 7C3).

In the iNon-treated Group, we observed in all analyzed animals remarkable smooth endoplasmic reticulum cisterns, ruptured mitochondria, and cristae loss, besides regions with more rarified cytoplasm in 7 dpir (Figure 7A1). At 30 and 60 dpir (Figures 7B1,C1), a rarer cytoplasm with an absence of organelles, ruptured mitochondria, and an absence of mitochondrial cristae was detected. A reduction of glycogen granules was also observed at 30 dpir.

### Prior Treatment With G-CSF Promotes Microenvironment Preservation

Immunostaining was evaluated for some of the main proteins related to normal liver tissue and some that are possibly involved in the regeneration and tissue recovery process promoted by treatment with G-CSF.

**FIGURE 8:**
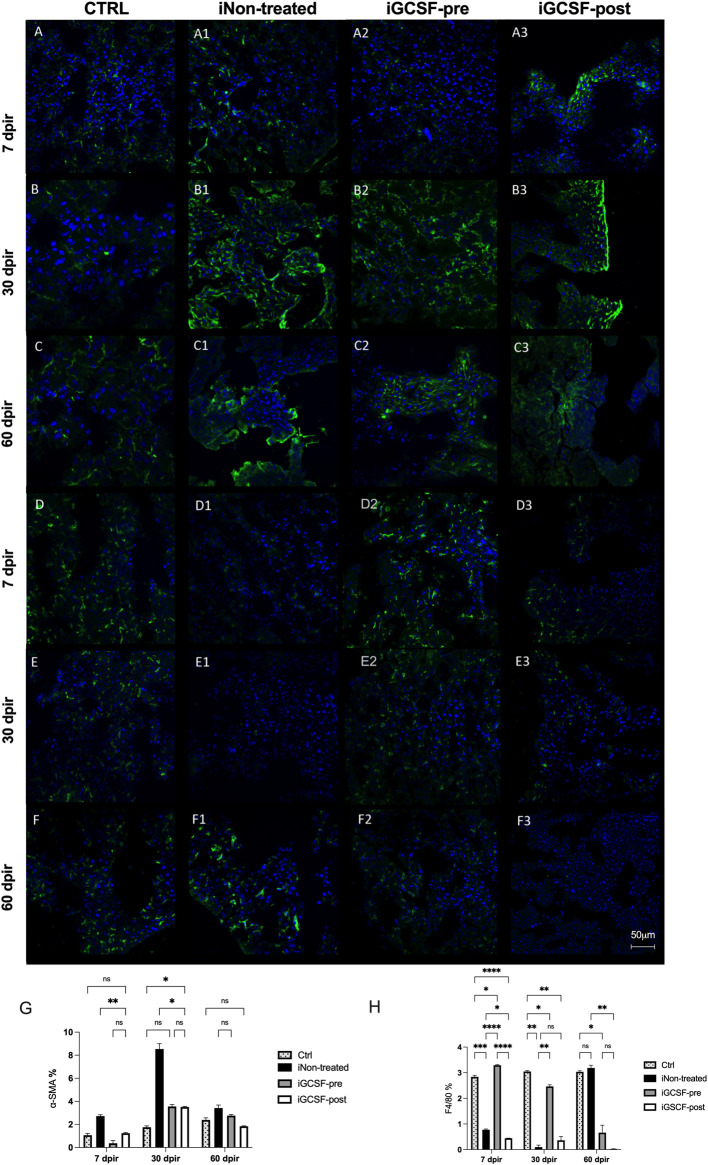
Liver tissue α-SMA and F4/80 protein expression evaluation. Representative images of α-SMA and F4/80 immunostaining from CTRL **(A–C,D–F)**, iNon-treated **(A1–C1,D1–F1)**, iGCSF-pre **(A2–C2,D2–F2)** and, iGCSF-post group **(A3–C3,D3–F3)** at 7, 30- and 60-day post-irradiation. Immunostaining images revealed positive cells for α-SMA with normal distribution and an increase in the α-SMA protein expression starting from 7 dpir **(A)**, which gradually increased to 60 dpir **(B–C)** at CTRL group. In the iGCSF-pre **(A2–C2)** group, this increase was observed in 30 dpir **(B2)**, decreasing slightly by 60 dpir **(C2)**. In the iGCSF-post Group **(A3–C3)**, this increase started at 7 dpir **(A3)** and continued to increase significantly at 30 and 60 dpir **(B3–C3)**. On the other hand, in the iNon-treated Group an increase was observed in 30 dpir **(B1)** and a slight decrease was observed in the iNon-treated Group **(C1)** at 60 dpir. Immunostaining images revealed F4/80 protein expression in the CTRL group **(D–F)**. In comparison to control, F4/80 protein expression was reduced in iNon-treated animals **(D1–F1)** in both 7 and 30 dpir **(D1–E1)**, but in 60 dpir **(F1)** the expression increased slightly, while in the group iGCSF-pre **(A2–F2)** this recruitment was increased since 7 dpir **(A2)** and was maintained until 60 dpir **(F2)**. A decrease in F4/80 protein expression was observed in 7, 30 and, 60 dpir in iGCSF-post **(D3–F3)** group. Quantification of cells expressing α-SMA **(G)** and F4/80 **(H)** in each group. The results are represented in mean ± sem. All groups were compared using Two-way analysis of variance followed by Tukey test, where *p < 0.05; **p < 0.01; ***p < 0.001 and ****p < 0.0001. Scale bars: 50 µm. Green = α-actin and F4/80, respectively, blue = DAPI (nuclei); (*n* = 5 per group).

To detect the activation of stellate cells, the expression of α-SMA was evaluated (Figure 8). At 7 dpir (*p* = 0.0116; *p* = 0.0284) and 30 dpir (*p* = 0.0134; *p* = 0.0066), the iGCSF-pre and iGCSF-post Groups showed a significant difference regarding α-SMA expression when compared to the iNon-treated Group. In the iGCSF-pre Group the α-SMA expression was similar to the CTRL group at 7 and 60 dpir (Figures 8A2,C2). On the other hand, in the iGCSF-post Group (Figures 8A3–C3), an increase in α-SMA expression started at 7 dpir (Figure 8A3) and continued to increase significantly at 30 dpir (*p* = 0.0188) and to decrease at 60 dpir (Figures 8B3–C3). In the iNon-treated Group, an increase was observed at 30 dpir (Figure 8B1) when compared to the iGCSF-pre and iGCSF-post Groups, and a significant decrease was observed at 60 dpir (Figure 8C1) (*p* = 0.0134)*.* In addition to the qualitative analysis, quantitative analysis was also performed and is shown in Figure 8G.

Furthermore, immunofluorescence analysis was performed to identify macrophages (F4/80) in the liver tissue (Figure 8). An increase in the recruitment of F4/80 cells was observed in animals previously treated with G-CSF at 7 dpir (*p* < 0.0001) in comparison to the iNon-treated and iGCSF-post Groups (Figure 8D2). However, a decrease was observed at 30 dpir (*p* = 0.0044) (Figure 8E2) and 60 dpir (*p* = 0.0447) in comparison to the control animals (Figures 8D–F). For the animals treated after irradiation (iGCSF-post), a reduction in the recruitment at 7 dpir (*p* = 0.0145; *p* < 0.0001) (Figure 8D3), which remained at 30 dpir (*p* = 0.0060; *p* = 0.0021) and 60 dpir (*p* = 0.0022; *p* = 0.0395) (Figures 8E3–F3), was observed when compared to the CTRL and iGCSF-pre Groups. A similar reduction was also observed in iNon-treated Group (Figures 8D1–E1), while an increase was detected at 60 dpir in this group (Figure 8F1). In addition to the qualitative analysis, quantitative analysis was also performed and is shown in Figure 8H.

**FIGURE 9:**
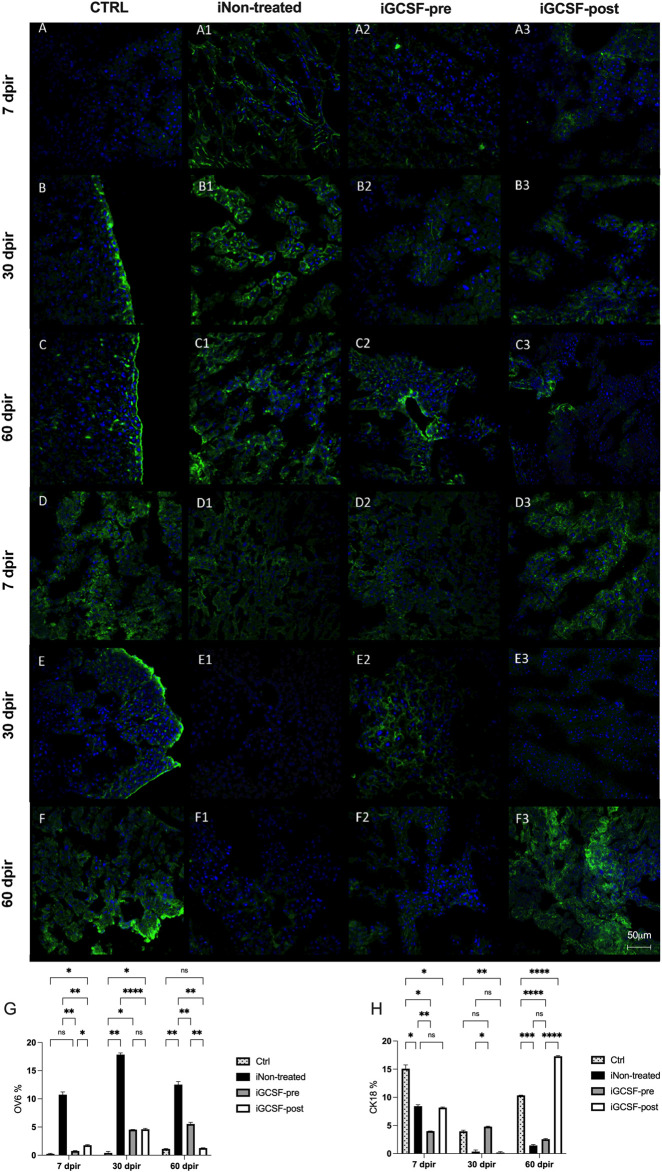
Liver stem cell (OV-6) and cytokeratin-18 (CK18) protein expression evaluation. Representative images of OV-6 and CK-18 immunostaining from CTRL **(A–C,D–F)**, iNon-treated **(A1–C1,D1–F1)**, iGCSF-pre **(A2–C2,D2–F2)** and iGCSF-post group **(A3–C3,D3–F3)** at 7, 30- and 60-day post-irradiation. Immunostaining images revealed low OV-6 protein expression in CTRL group **(A–C)**. In iNon-treated Group **(A1–C1)**, an increase in OV-6 protein expression was observed at all analyzed times. In iGCSF-pre Group **(A2–C2)**, a remarkable increase in OV-6 protein expression was observed. Similarities regarding OV-6 protein expression between iGCSF-post Group **(A3–C3)** and iNon-treated animals were identified. Immunostaining images revealed a normal CK-18 protein expression distribution in CTRL group **(D–F)**. In comparison to the control group, a decrease in CK-18 protein expression was observed from 30 dpir **(E1)**, which remained until 60 dpir **(F1)** in the iNon-treated Group. Immunoreactivity similar to the control was observed between groups iGCSF-pre **(D2–F2)** and iGCSF-post **(D3–F3)** groups. A remarkable increase in CK-18 protein expression was observed in iGCSF-pre Group in 30 **(E2)** and 60 dpir **(F2)** in comparison to iGCSF-post Group **(E3–F3)**. Quantification of Liver stem cell (OV-6) **(G)** and cytokeratin-18 (CK18) **(H)** in each group. The results are represented in mean ± sem. All groups were compared using Two-way analysis of variance followed by Tukey test, where *p < 0.05; **p < 0.01; ***p < 0.001 and ****p < 0.0001. Scale bars: 50 µm. Green = OV-6 and CK-18, respectively, blue = DAPI (nuclei); (*n* = 5 per group).

To investigate if the G-CSF treatment could promote liver regeneration in our experimental model, we also performed immunofluorescence analysis to identify positive hepatic oval cells using the oval-cell 6 markers (OV-6). This protein is considered a marker of liver stem cells. Compared to the CTRL Group (Figures 9A–C), a significant increase in OV-6 expression was observed in the iGCSF-pre Group at 30 dpir (*p* = 0.0016) and 60 dpir (*p* = 0.0069) (Figures 9A2–C2). In the iGCSF-post Group (Figures A3–C3), OV-6 expression started to increase at 7 dpir (*p* = 0.0421) and continued to increase significantly at 30 dpir (*p* = 0.0260), decreasing at 60 dpir compared to the CTRL Group. A significant increase of OV-6 expression was observed in the iNon-treated Group (Figures 9A1–C1) at all time points when compared to the CTRLand iGCSF-pre and post-Groups. In addition to the qualitative analysis, quantitative analysis was also performed and is shown in Figure 9G.

We also evaluated CK18 positive cells by immunofluorescence analysis. The liver tissue of the CTRL Group (Figures 9D–F) showed a normal CK18 positive cell distribution. In the iNon-treated Group (Figures 9D1–F1), we observed a decrease in CK18 protein expression at 7 dpir (*p* = 0.0101) (Figure 9E1). Also, a remarkable decrease in CK18 protein expression was observed at 30 dpir (*p* = 0.0149) and 60 dpir (*p* = 0.0002) (Figure 9F1) compared to the CTRL Group. In the iGCSF-pre Group (Figures 9D2–F2), we observed a decrease in CK18 protein expression at 7 dpir (*p* = 0.0110 and 60 dpir (*p* < 0.0001) when compared to the CTRL Group. In the iGCSF-post Group (Figures 9D3–F3) there was a decrease at 7 dpir (*p* = 0.0243)*,* which was accentuated at 30 dpir (*p* = 0.0041) and reversed at 60 dpir (*p* < 0.0001) compared to the CTRL Group. In addition to the qualitative analysis, quantitative analysis was also performed and is shown in Figure 9H.

Finally, we performed immunostaining analysis to detect alpha-fetoprotein (AFP) and cytokeratin 19 protein expression. However, we did not detect AFP and CK19 protein expression in all analyzed tissues (Supplementary Figure S1).

### Prior Treatment With G-CSF Improves Survival Rates

**FIGURE 10:**
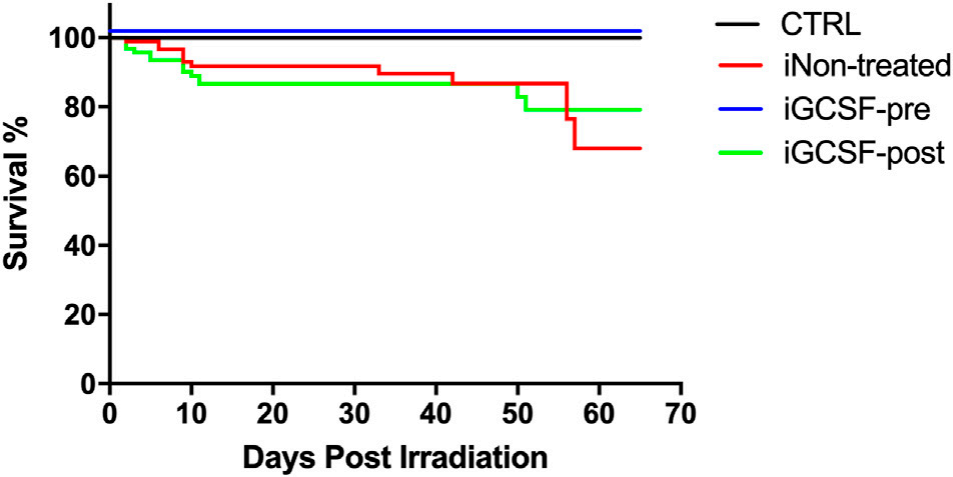
Survival analysis. Kaplan–Meier curves of survival for 60-day post-irradiation in the CTRL, iNon-treated, iGCSF-pre and iGCSF-post Group (*n* = 20 per Group). The survival is statistically significant in a log-rank (Mantel-Cox) test, where the comparison of all survival curves ****p < 0.0001; iGCSF-pre vs. iGCSF*-*post ***p = 0.0002; iGCSF-pre vs. iNon-treated ***p = 0.0005 and iGCSF*-*post vs. iNon-treated *p* = 0.6929.

A group of animals that went through all the procedures but was not euthanized in any of the evaluated time points was used to estimate the survival rates between the different groups using the Kaplan-Meier curve. Sixty days after irradiation, the survival rate of the animals previously treated with G-CSF (iGCSF-pre, *n* = 20) was equal to the CTRL Group (100%), with no deaths until the end of the protocol. However, the iNon-treated (*n* = 20) and iGCSF-post (*n* = 20) survival rates were 68 and 79%, respectively (Figure 10). The survival was statistically significant in a log-rank (Mantel-Cox) test, where the comparison of all survival curves ****p < 0.0001; iGCSF-pre vs. iGCSF*-*post ***p = 0.0002; iGCSF-pre vs. iNon-treated ***p = 0.0005 and iGCSF*-*post *vs.* iNon-treated *p* = 0.6929.

## Discussion

In the present study, we evaluated the benefits of G-CSF administration before and after irradiation exposure. A new radiation-induced liver disease (RILD) experimental model based on the association of long-term alcohol administration and irradiation exposure was established. To investigate our hypothesis, 4-week-old male and female C57BL/6 mice were assigned to four groups: the CTRL Group; the iNon-treated Group, which was irradiated locally in the liver with 18 Gy; the iGCSF-pre Group which was treated with G-CSF (100 μg/kg/day) subcutaneously during the 3 days preceding radiotherapy; and the iGCSF-post Group, which received the same treatment only during the 3 days following radiotherapy. All groups were given ad libitum access to water (CTRL) or alcohol diluted (5%) in water for 3 months and were analyzed 7, 30 and, 60 days after irradiation (dpir). We combined non-invasive methods (high-resolution ultrasound analysis, magnetic resonance imaging, and computed tomography analysis) with conventional quantitative and qualitative analysis to address the histological, biochemical, and immunohistology factors improved by the G-CSF administration. Our data showed that the treatment with G-CSF before irradiation effectively improved morphofunctional parameters caused by RILD, restoring histological arrangement and promoting liver regeneration and survival rates increases.

Clinically, radiotherapy reduces tissue flexibility, inducing senescence and/or cell death, loss of stem cells, late vascular damage, and chronic organ and tissue dysfunction (De Ruysscher et al., 2019). These specifics adverse effects can occur in different organs during irradiation exposition, including the brain, promoting alterations in the central nervous system that result in metabolic syndrome and liver fibrosis development via the liver-brain inflammation axis (Bentzen, 2006).

It is well known that RILD is responsible for high mortality rates during radiotherapy treatment in humans. Similarities were described when different experimental models were exposed to irradiation to mimic the human RILD (Du et al., 2010; Rave-Fränk et al., 2013; Yannam et al., 2014; Kim and Jung, 2017). The irradiation doses required to cause liver damage in mice are lethal after a few days, making analysis impossible later. Several reports described a variation in irradiation dose used to promote RILD in mice (Wang et al., 2013a; Wang et al., 2013b; Wang et al., 2013c). According to Wang et al. (Wang et al., 2013a), 70% mortality for 1 week and 100% for 10 days were showed when mice were irradiated with a 20 Gy dose. We established an efficient protocol to promote alcohol-induced liver injury and irradiation exposure association to overcome this problem. The combination of these chemical and physical agents was efficient in reproducing human RILD pathogenesis. In addition, we established a similar human RILD using a previous liver injury developed by 90-day alcohol administration in association with a non-lethal irradiation dose.

In contrast with the reported by Wang et al. (2013a), our protocol showed a 68% mortality rate for 8 weeks when mice were exposed to an 18 Gy irradiation dose. Also, the pre- and post-treatment with G-CSF improved the survival rate. More importantly, our results showed that all mice receiving G-CSF administration before the irradiation challenge survived for 60 days post-irradiation. This increase in survival rates was associated with G-CSF treatment.

It has been proposed that G-CSF, an agent commonly used for neutropenia treatment, can stimulate hematopoietic cell mobilization to the injured liver, facilitating liver regeneration and consequently improving survival rates (Liu et al., 2006; Li et al., 2010; Garg et al., 2012; Duan et al., 2013; Chavez-Tapia et al., 2015). In this study, we aimed to investigate whether the G-CSF could be able to promote hepatic protection post-irradiation exposition. To support our hypothesis, morphological, biochemical, and ultrastructural analyses were performed. Our data suggest that G-CSF administration before irradiation ameliorates the histological damage, preserving mitochondria, improving liver function, and restoring serum biochemical parameters to regular rates. In addition, non-invasive methods analysis were applied to understand the benefits promoted by G-CSF administration. High-resolution ultrasonography and magnetic resonance imaging revealed that G-CSF administration before irradiation effectively promoted liver homogeneous echogenicity parameters, a regular surface, and contours maintaining an intense and homogeneous signal. Considering the echogenicity parameters reported by Lessa et al. (2010), normal liver parenchyma was found when the G-CSF was administrated before irradiation exposure. In agreement, these findings confirm our hypothesis that G-CSF could act on the liver to promote its recovery.

Wang et al. (2013a) reported fat accumulation into hepatocytes after irradiation exposure. To investigate the differences between the treatment with G-CSF before or after irradiation exposure, computed tomography analysis was performed to assess the level of fat infiltration. Our results showed that it was more intense in untreated animals than animals treated with G-CSF, demonstrating that G-CSF plays a role in reducing the infiltration generated by liver damage. Besides fatty accumulation, according to Liang et al. (2018), the RILD is remarkable by liver fibrosis, a pathological response to inflammation and tissue damage promoted by irradiation exposure. Therefore, after assessing fat infiltration, we investigated if the treatment with G-CSF was able to ameliorate the hepatic fibrosis promoted by RILD. The histological assessment of reticulin fibers showed that the G-CSF administration before irradiation alleviates liver fibrosis development. On the one hand, the anti-fibrotic effects reported here were in consonance with the results previously reported by Tsolaki et al. (2014). However, we observed that the RILD generated an increase in reticulin fibers in the liver tissue, but G-CSF could not prevent this accumulation completely when administrated before irradiation or reverse it when administrated after irradiation. In this study, as reported in the literature (Yuan et al., 2019) we observed an increase in protein expression of α-SMA after irradiation. Besides, the administration of G-CSF after the irradiation exposure was not able to revert this condition. However, the treatment with G-CSF before irradiation promoted a delay in the appearance of α-SMA positive cells, probably to avoid liver injury and fibrosis, since the expression of α-SMA cells in this group only increased after 30 days post-irradiation. Furthermore, the previous treatment with G-CSF was efficient in decreasing hepatic stellate cell activation, a typical result of radiation exposure. Also, the previous treatment with G-CSF contributed to alleviating liver tissue damage promoted by RILD. Other cell markers were studied to elucidate G-CSF’s tissue preservation mechanisms, and we observed that the previous G-CSF administration promoted similarities to normal tissue regarding F4/80 positive cells after irradiation. However, Higashiyama et al. reported a small F4/80 cell population in the liver when G-CSF was used in hepatic fibrosis induced by carbon tetrachloride in a mice model (Higashiyama et al., 2007). Besides, the G-CSF preserved the amount of CK18 positive cells in irradiated liver tissues. In general, we observed that the previous G-CSF administration promoted liver tissue homeostasis, maintaining a normal cell population.

In opposition to Li et al. (2010), our data indicated the administration of G-CSF before irradiation protected the liver from RILD. We hypothesized that G-CSF acted as a hematopoietic and hepatic stimulator, promoting cell mobilization and efficient hepatic microenvironment arrangement to respond to radiation exposure. It is possible that when the hepatocytes are unable to initiate the regeneration of an injury or when the injury is severe, populations of intrahepatic stem cells called liver progenitors or oval cells act to promote liver regeneration and hepatic tissue healing (Piscaglia et al., 2007). This study indicated that under the injury process, the oval cells are activated and contribute to regenerative responses, giving rise to hepatocytes and other non-parenchymal cells.

Consequently, a greater expression of OV-6 was found in the injured and untreated group. According to Awan et al. (2017), the number of oval cells is correlated with the severity of the liver injury. This was clear when we observed non-treated animals.

Based on our findings, we observed that treatment with G-CSF effectively promoted liver tissue homeostasis, protecting against liver fibrosis development and restoring the liver arrangement, preserving liver cell population and hepatic functions after irradiation exposure. However, the molecular mechanisms need to be further studied. In summary, G-CSF exerts multiple direct and indirect immune and hepatic effects able to promote liver protection. Furthermore, the present results showed that the previous treatment with G-CSF prevented and alleviated the liver injury process promoted by 90-day alcohol-induced liver disease and radiation exposure. Therefore, G-CSF can be potentially used in the treatment of RILD patients in the future to prevent disease development and low survival rates.

## Data Availability

The original contributions presented in the study are included in the article/Supplementary Material, further inquiries can be directed to the corresponding author.
