# Supplementary material for: Granulocyte Colony-Stimulating Factor Treatment Before Radiotherapy Protects Against Radiation-Induced Liver Disease in Mice
Source: Front Pharmacol. 2021 Nov 15;12:725084. doi: 10.3389/fphar.2021.725084 (PMC8634713; doi:10.3389/fphar.2021.725084)
Supplement: Supplementary file 1 [file Table1.pdf]

Granulocyte Colony-Stimulating Factor treatment before radiotherapy protects against Radiation-Induced liver disease in mice

Isalira Peroba Rezende Ramos[1], Marlon Lemos Dias[2,3], Alan Cesar Nunes de Moraes[4], Fernanda Guimarães Meireles Ferreira[5], Sergio Augusto Lopes Souza[6], Bianca Gutfilen[6], Thiago Barboza[6], Cibele Ferreira Pimentel[2,3,7,8], Cintia Marina Paz Batista[2], Tais Hanae Kasai-Brunswick[1,2,3], Fabio da Silva de Azevedo Fortes[7], Cherley Borba Vieira de Andrade[2,8], Regina Coeli dos Santos Goldenberg[2,3]

1Centro Nacional de Biologia Estrutural e Bioimagem- CENABIO, Universidade Federal do Rio de Janeiro, UFRJ, Rio de Janeiro, Brasil,

2Instituto de Biofísica Carlos Chagas Filho, Universidade Federal do Rio de Janeiro, UFRJ, Rio de Janeiro, Brasil,

3Instituto Nacional de Ciência e Tecnologia em Medicina Regenerativa, INCT-REGENERA, Universidade Federal do Rio de Janeiro, UFRJ, Rio de Janeiro, Brasil,

4Departamento de Biologia, Universidade Federal Fluminense, UFF, Niterói, Brasil,

5Instituto D'Or de Pesquisa e Educação, Rio de Janeiro, Brasil,

6Departamento de Radiologia, Hospital Universitário Clementino Fraga Filho, Universidade Federal do Rio de Janeiro, Rio de Janeiro, Brasil,

7Laboratório de Terapia e Fisiologia Celular e Molecular- LTFCM, Centro Universitário Estadual da Zona Oeste-UEZO, Rio de Janeiro, Brasil,

8Programa de Pós-Graduação em Biomedicina Translacional- BIOTRANS (UEZO-UNIGRANRIO-InMETRO), Brasil,

9Departmento de Histologia e Embriologia, Universidade do Estado do Rio de Janeiro, UERJ, Rio de Janeiro, Brasil

***Corresponding author:** Regina Coeli dos Santos Goldenberg, PhD (rcoeli@biof.ufrj.br), Carlos Chagas Filho Biophysics Institute, Federal University of Rio de Janeiro, Rio de Janeiro, Brazil. Avenida Carlos Chagas Filho, 373, Ilha do Fundão, Rio de Janeiro, RJ, 21941-902, Telephone: +55 21 99991-3066.

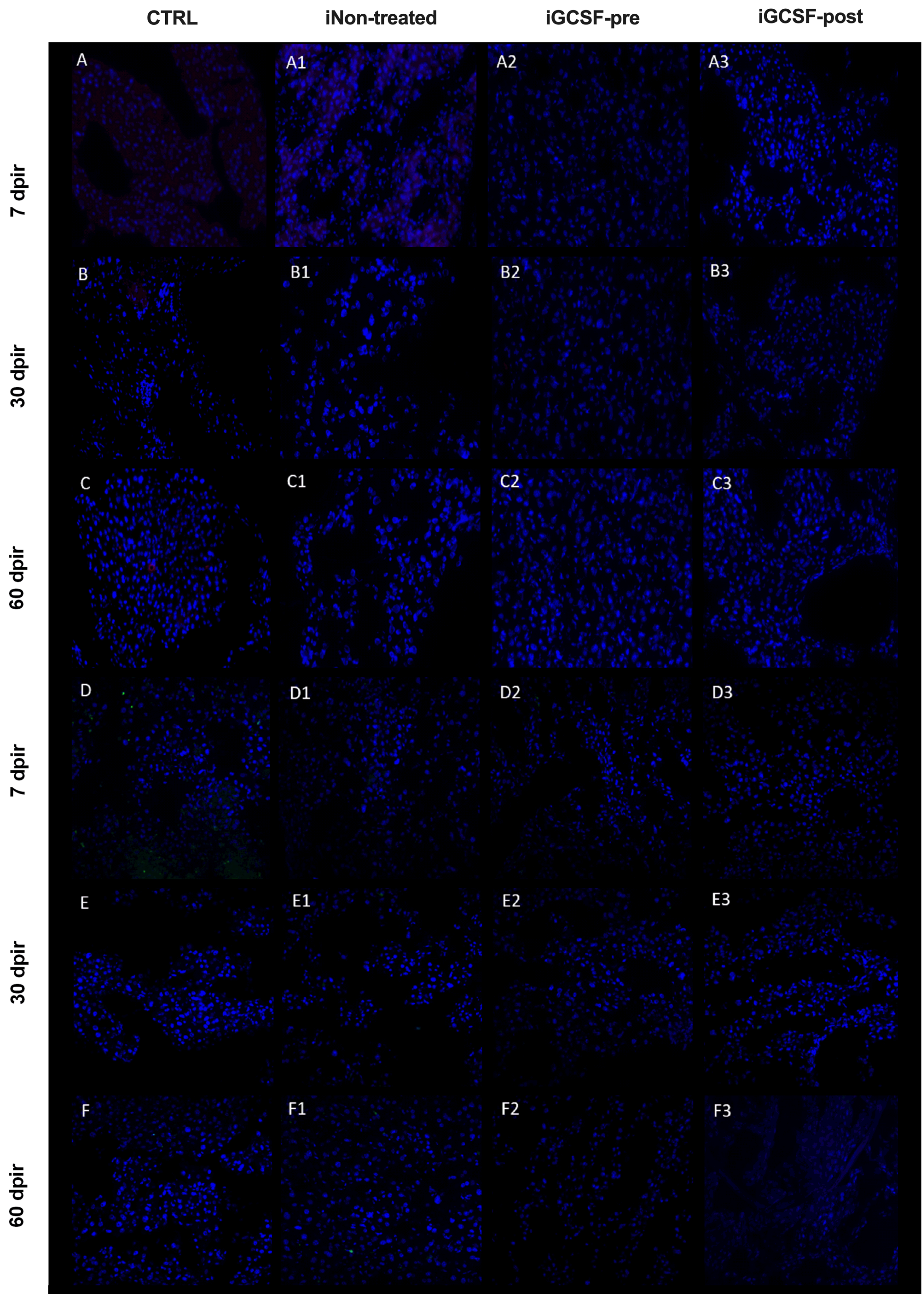

Supplementary Figure 1. Liver tissue CK-19 and AFP protein expression evaluation. Representative images of CK-19 and AFP immunostaining from CTRL (A-C; D-F), iNon-treated (A1-C1; D1-F1), iGCSF-pre (A2-C2; D2-F2), and iGCSF-post group (A3-

C3; D3-F3) at 7, 30- and 60-days post-irradiation. No positive immunostaining was observed. Scale bars: 50µm. Green= CK-19 and AFP, respectively, blue= DAPI (nuclei).
